# The Challenges of Diagnosis and Treatment of Arrhythmogenic Cardiomyopathy: Are We there yet?

**DOI:** 10.31083/j.rcm2308283

**Published:** 2022-08-15

**Authors:** Alberto Spadotto, Domenico Morabito, Alessandro Carecci, Giulia Massaro, Giovanni Statuto, Andrea Angeletti, Maddalena Graziosi, Elena Biagini, Cristian Martignani, Matteo Ziacchi, Igor Diemberger, Mauro Biffi

**Affiliations:** ^1^IRCCS Azienda Ospedaliero-Universitaria di Bologna, 40138 Bologna, Italy; ^2^Department of Experimental, Diagnostic and Specialty Medicine, 40138 Bologna, Italy

**Keywords:** arrhythmogenic cardiomyopathy, diagnosis, treatement, ICD therapy

## Abstract

**Background:**

we sought to review the evolution in the diagnosis and treatment of Arrhythmogenic Cardiomyopathy (ACM), a clinically multifaceted entity beyond the observation of ventricular arrhythmias, and the outcome of therapies aiming at sudden death prevention in a single center experience.

**Methods:**

retrospective analysis of the data of consecutive patients with an implanted cardioverter-defibrillator (ICD) and a confirmed diagnosis of ACM according to the proposed Padua Criteria, who were referred to our center from January 1992 to October 2021.

**Results:**

we enrolled 72 patients (66% males, mean age at implant 46 ± 16 years), 63.9% implanted for primary prevention. At the time of ICD implant, 29 (40.3%) patients had a right ventricular involvement, 24 (33.3%) had a dominant LV involvement and 19 (26.4%) had a biventricular involvement. After a median follow-up of 6,1 years [IQR: 2.5–9.9], 34 patients (47.2%) had 919 sustained episodes of ventricular arrhythmias (VA). 27 patients (37.5%) had 314 episodes of life-threatening arrhythmias (LT-VA), defined as sustained ventricular tachycardia ≥200 beats/min. Considering only the patients with an ICD capable of delivering ATP, 80.4% of VA and 65% of LT-VA were successfully terminated with ATP. 16 (22.2%) patients had an inappropriate ICD activation, mostly caused by atrial fibrillation, while in 9 patients (12.5%) there was a complication needing reintervention (in 3 cases there was a loss of ventricular sensing dictating lead revision). During the follow-up 11 (15.3%) patients died, most of them due to heart failure, and 8 (11.1%) underwent heart transplantation.

**Conclusions:**

ACM is increasingly diagnosed owing to heightened suspicion at ECG examination and to improved imaging technology and availability, though the diagnostic workflow is particularly challenging in the earliest disease stages. ICD therapy is the cornerstone of sudden death prevention, albeit its efficacy is not based on controlled studies, and VT ablation/medical therapy are complementary to this strategy. The high burden of ATP-terminated VA makes shock-only devices debatable. The progressive nature of ACM leads to severe biventricular enlargement and refractory heart failure, which pose significant treatment issues when a predominant RV dysfunction occurs owing to the reduced possibility for mechanical circulatory assistance.

## 1. Introduction

**Fig. 1.**
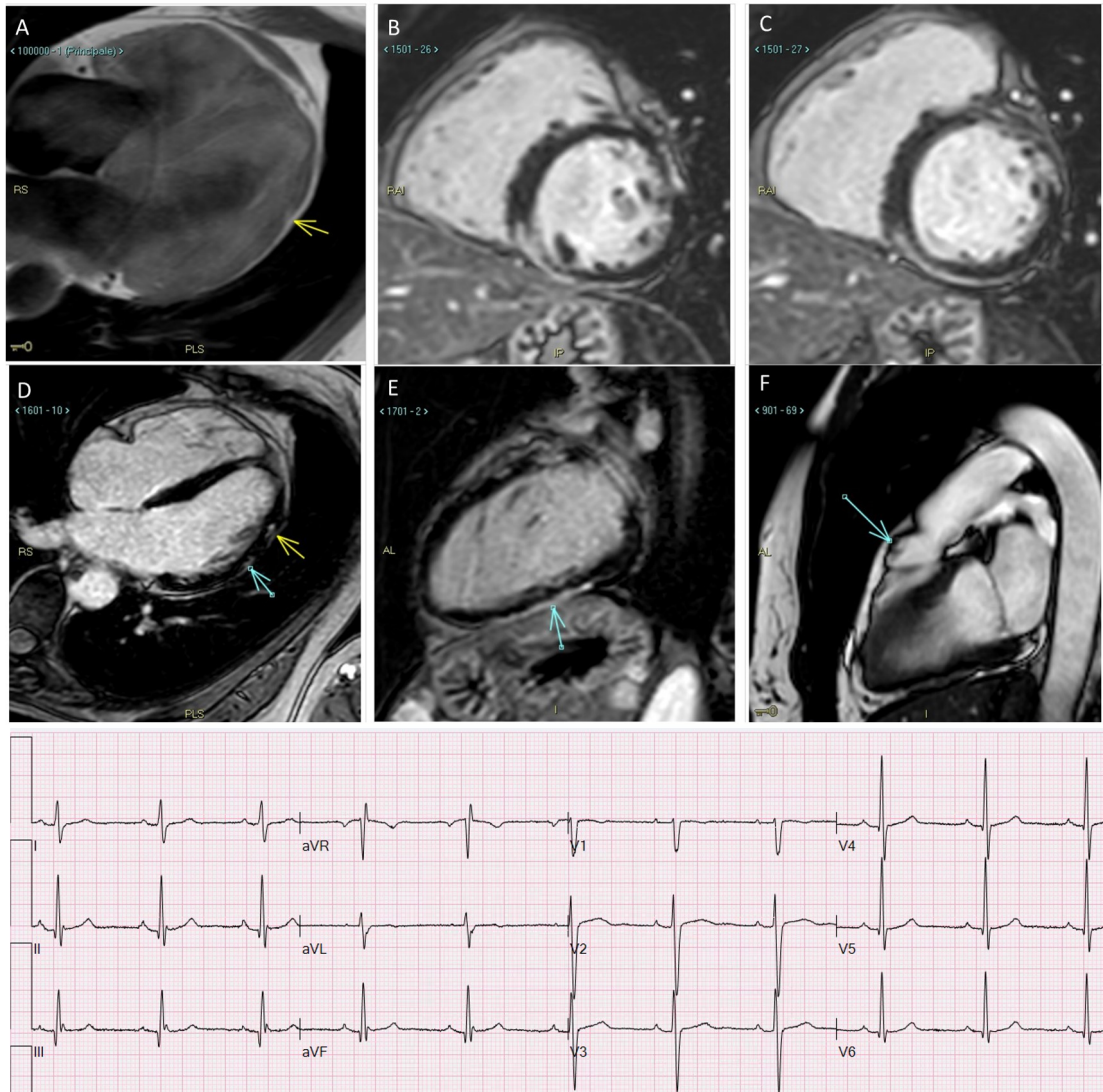
**Late-onset biventricular ACM in a 54-years old lady with self-terminating syncopal ventricular tachycardia, screened at 44 as mother of an ALVC proband, both with desmoplakin mutation**. While ECHO and ECG were normal at 44, CMR at 54 shows: (A) fibrofatty infiltration located in subepicardic lateral wall of the LV. (B,C) Ring-like LGE located in the inferior wall and in the inferior interventricular septum. (D) Lateral wall focal areas of LGE and mild RV enlargement with anterior hypokinesia. (E) Inferior wall LGE. (F) RVOT bulging. See fragmented QRS mimicking a pseudo-epsilon wave in inferior limbs (negative in aVL) at ECG.

**Fig. 2.**
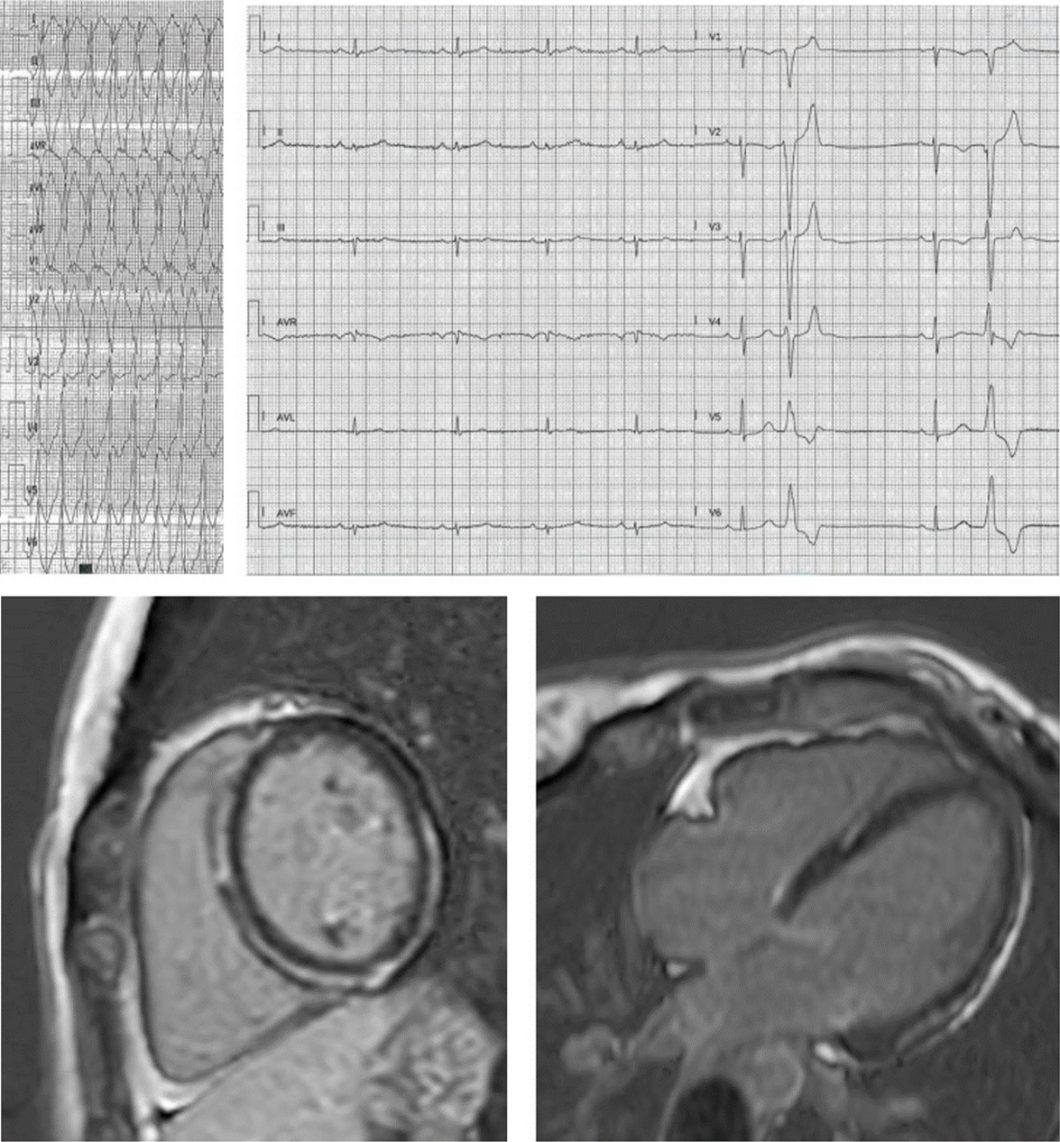
**Proband, aged 27 years, desmoplakin mutation: poorly tolerated monomorphic VT during training**. See a midlayer ring-like fibrotic tissue involvement of the LV, without RV involvement. ECG shows low-amplitude limb leads with multifocal ventricular beats stemming from the anterior LV wall.

**Fig. 3.**
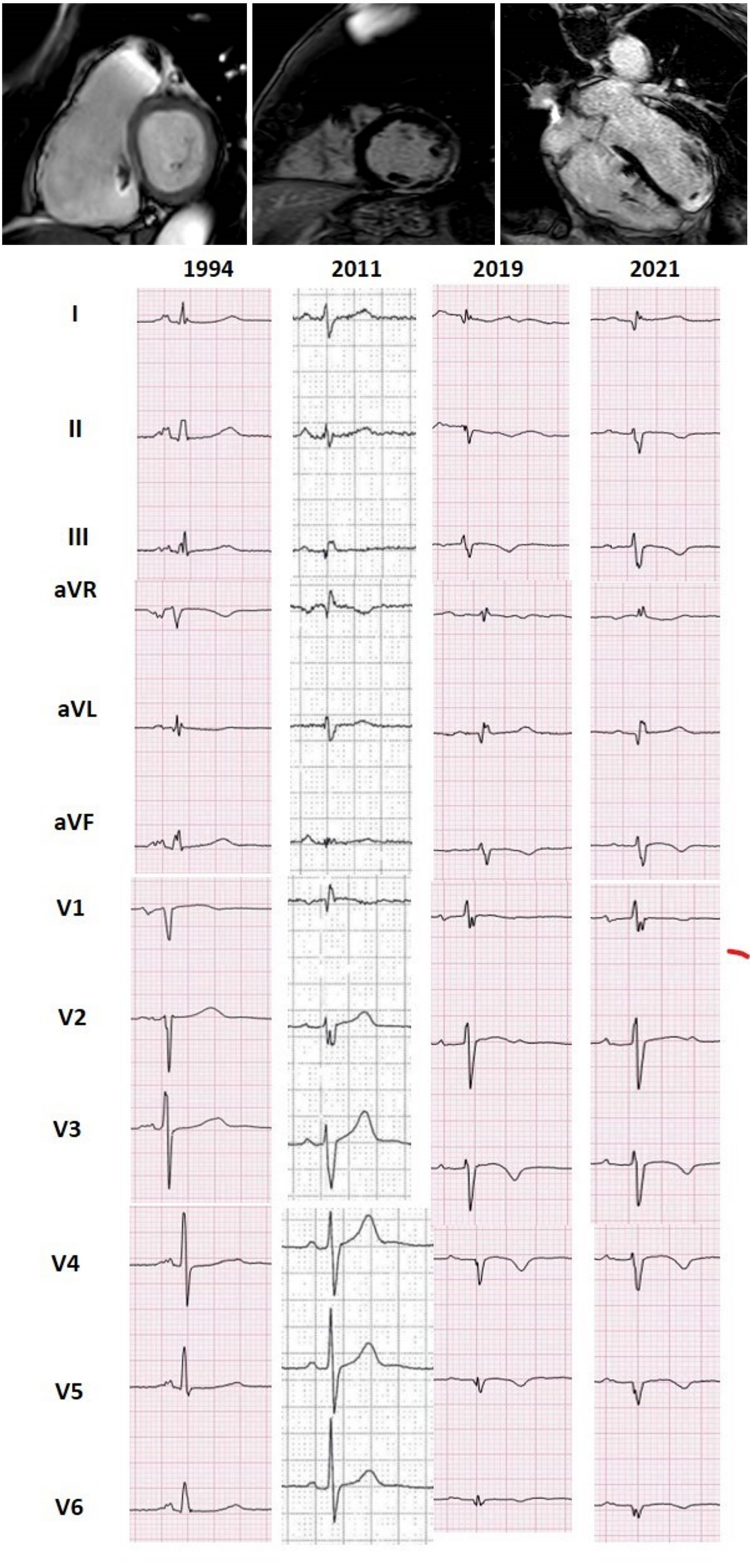
**Progressive fibrofatty replacement of the RV and later of the LV in a patient with PKP2 mutation, detected by serial ECG recordings (A) from age 44 up to 72, implanted in secondary prevention at 66 because of monomorphic VT at 210 bpm**. Both ECG and CMR (B) show epicardial, midventricular and also transmural fibrotic involvement of the inferior and the posterior lateral LV wall, mimicking ischemic cardiomyopathy in the absence of coronary artery disease. The RV involvement and the progressive ECG changes along years (transition from a normal pattern to an RV and eventually to extensive LV involvement) hinted at genetic testing for the etiologic diagnosis.

This review on Arrhythmogenic Cardiomyopathy (ACM) focuses on its diagnostic challenges, on the debated role of risk-stratification of sudden cardiac death, and reports the outcome of ICD treatment for sudden death prevention in all cardiac phenotypes. ACM is a general term that encompasses a group of diseases different amongst themselves depending on type of pathologic involvement of the heart, aetiology, and genetics. While it is rationale to assign a specific nosographic classification to entities having a homogeneous genetic background (mutations of the same gene/group of genes) resulting in a common clinical phenotype (Figs. 1,2), it is much more clinically challenging to classify a disease whose phenotypic appearance is the outcome of several unlinked genetic diseases with different pathogenic mechanisms. Even more difficult is disease classification when different mutations of the same gene are disease-causative in the same organ with different phenotypes (hypertrophic vs arrhythmogenic disease). Debate as to whether a genomic-based classification of diseases or a clinical phenotype-based one is more appropriate is ongoing. We focus on ACM phenotype expressed as right ventricular, biventricular, and left ventricular involvement of the heart caused by progressive replacement of the myocardium by fibrotic or fibro-fatty tissue, which acts as an arrhythmogenic substrate predisposing to life-threatening ventricular arrhythmias and heart failure due to systolic ventricular dysfunction, caused by inherited genetic abnormalities. The earliest clinical manifestations of these diseases are ventricular arrhythmias, typically occurring between the third and the fourth decade, though they represent a relevant cause of sudden death in adolescents, especially in the physically active and in high-level athletes [1]. Sudden cardiac death (SCD) can be the first manifestation in a minority of patients, thereby heightening medical attention in the event ventricular arrhythmias are detected in otherwise healthy and young individuals. Progressive fibrotic replacement of myocardial cells leads to ventricular dysfunction and heart failure (Fig. 3) in the advanced stages of the disease, which can have an extremely different time course across individuals. Several efforts aimed at understanding, diagnose, and manage ACM have been made in the past decades, yet there is an ongoing debate surrounding ACM.

## 2. Pathogenesis and Genetic Aspects

### 2.1 Pathophysiology of Desmosomal Abnormalities

Firstly considered a congenital malformation, nowadays it is known that ACM is a genetically inherited disease, in most cases autosomally dominant, which develops after birth. This knowledge stems from the recognition of two recessively inherited cardiocutaneous syndromes, namely Naxos [2] and Carvajal [3] diseases, whose affected individuals share a common phenotype characterized by palmoplantar keratoderma, woolly hair and arrhythmogenic right ventricular cardiomyopathy. The identification of these syndromes led to the discovery of the responsible mutated genes: plakoglobin (JUP) for Naxos disease and desmoplakin (DSP) for Carvajal disease.

Plakoglobin and desmoplakin are components of a transmembrane complex named desmosome. Other elements include desmoglein-2 (DSG2) [4], desmocollin-2 (DSC2) [5] and plakophilin-2 (PKP2) [6]. As its name suggests (“binding body”), the role of the desmosome is to mediate adhesion between cells. However, it also has much more complex functions such as anchoring cytoskeleton to cell membrane, intracellular signaling and electrical coupling by organizing gap junctions and ion channels [7].

In the context of altered desmosome structure and function, cardiomyocytes are prone to loose adhesion to each other [8], a process that is amplified by mechanical stress. This leads to altered intracellular signaling with suppression of the Wnt/β-catenin pathway resulting in apoptosis and up-regulation of adipogenesis transcriptional factors. Plakoglobin shares structural and functional properties with β-catenin. In cultured DSP-deficient atrial myocytes [9] and heterozygous cardiac-specific DSP-knockout mouse models [10], it shows increased nuclear translocation where it can compete with β-catenin for binding to transcription factors, resulting in decreased expression of Wnt target genes (c-Myc and cyclin-D1). Interestingly, inhibition of glycogen synthase kinase-3 beta (GSK-3β), which targets β-catenin for degradation, reversed desmosomes and gap junctions remodeling and prevented cardiac dysfunction [11].

Desmosome interacts with connexin 43 (Cx43) and sodium channel ${\mathrm{Na}}_{v}$1.5 possibly implying a role in arrhythmogenesis. Heterozygous knockout PKP2 and DSP mice models have indeed shown altered sodium currents kinetics and induction of ventricular arrhythmias without overt histological alterations [12].

### 2.2 Non-Desmosomal Abnormalities in ACM

Mutations of desmosome components are the most commonly observed in ACM patients, with PKP2 being the most frequent. Nonetheless, a wealth of mutations also in non-desmosomal proteins have been described:

- N-Cadherin (CDH2) is a transmembrane adherens junction which provides calcium-dependent adhesion, connects actin filament between sarcomeres, stabilises gap junctions and has many roles in embryogenesis. In 2017 a novel variant of CDH2 was found in a South African ARVC-affected family [13].

- Lamins are nuclear intermediate filament proteins, encoded in the LMNA gene, that have suppressive effects on the expression of many genes [14]. Pathogenic variants of LMNA have been described in a variety of cardiac manifestations: atrial fibrillation [15], conduction disease, ventricular arrhythmias and dilated cardiomyopathy [16, 17]. Its role in ACM is not completely understood, though. The high incidence of SCD in these families has led to recommendations to consider prophylactic implantable cardioverter defibrillators (ICD) for SCD prevention [18].

- Desmin (DES) is a muscle-specific intermediate filament protein which is involved in linking Z-disk to nuclear and cellular membranes, sarcomere synthesis, nuclear positioning, sarcoplasmic reticulum and T-tubular system. DES mutations have been reported in all phenotypes of cardiomyopathy as well as skeletal myopathies, but they are frequently associated to DCM typically exhibiting a high incidence of conduction system disease and arrhythmias [19, 20]. ACM DES-mutated carriers have been described to show a fully penetrant variable cardiac phenotype with a propensity for left ventricular involvement [21].

- Filamin C (FLNC) in an actin cross-linking protein found in striated muscle cells. Truncating variants of this gene have been associated with a left dominant ACM [22] with an elevated risk of SCD. Other variants have been reported in restrictive [23] and hypertrophic [24] cardiomyopathies.

- Transmembrane 43 is a nuclear membrane protein known to bind with Lamin and other nuclear proteins. Mutations of this protein, firstly described in Newfoundland, have been found to cause a fully penetrant biventricular ACM [25, 26] with a substantial risk for SCD in males.

- Phospholamban (PLN) is a sarcoplasmic homopentameric protein involved in calcium homeostasis by regulating the activity of sarcoplasmic reticulum ${\mathrm{Ca}}^{2+}$- ATPase (SERCA). PLN mutations make its product unable to be inactivated through cAMP-protein kinase mediated phosphorylation [27]. In the unphosphorylated state, phospholamban inhibits SERCA-mediated ${\mathrm{Ca}}^{2+}$ re-uptake back in the sarcoplasmic reticulum. Thereby, cytosolic calcium increases, which is known to cause delayed after depolarization (DAD), possibly triggering arrhythmias. PLN p.R14del mutation carriers are quite common in the Netherlands (1 in 1500 Dutch people). In 2012 a cohort a DCM and ARVC were screened for this specific mutation, and it was found in 15% and 12% respectively [28]. Affected individuals show a variable phenotype with right, left, or biventricular involvement. The high incidence of SCD in affected individuals led to ICD implant recommendation similar to LMNA-related cardiomyopathy.

- Voltage-gated sodium channel (encoded by SCNA5) mutations are known to cause Brugada syndrome (loss of function) and type 3 long QT syndrome (gain of function). Both types of mutations can lead to DCM [29] with no apparent fibrosis and frequent conduction disease and ventricular arrhythmias. However, it is not clear whether the DCM phenotype is due to genetic defect or consequent to frequent ventricular arrhythmias.

- Mutations of Titin (TTN), the biggest human protein and essential component of sarcomere, have been reported in 7 out of 38 families suffering from ARVC with negative desmosomal genetic testing [30]. The clinical history was characterised by high penetrance (86%) with SCD, severe myocardial dysfunction leading to death or heart transplantation, and conduction system disease. TTN nonsense mutations are also the first genetic cause of DCM [31, 32].

- RBM20 encodes an RNA-binding protein involved in constitutive and alternating splicing of key cardiac genes including sarcomeric proteins and ion regulating proteins. Loss of function mutations leads to missplicing of these proteins. Affected patients present with an early-onset and fastly-progressing disease with severe heart failure, high arrhythmic burden and SCD [33, 34].

Other implicated genes with limited evidence are CTNNA3, TJP1, RYR2, NKX2-5, BAG3, TGFβ3 [35].

Genetic mutations, though important, cannot explain the entire disease pathogenesis. In fact, they can be found in about 60% ACM patients, with desmosomal genes accounting for the majority of them [36]. Owing to the development of next generation sequencing and of genome-wide association studies, variants of potential culprit genes keep being discovered and reported, but in many cases their pathogenicity is of uncertain significance. On the other hand, pathogenic variants can be found in healthy controls and relatives of affected individuals who show no signs of the disease. Other factors are involved in the development and progression of the disease, for instance mutations of genes encoding factors limiting disease expression. Patients with multiple mutations (compound heterozygosity, digenic heterozygosity) are known to have a higher penetrance and an earlier onset of disease [37], but their frequency is low, ranging from 4% to 10%.

Genetic tests for ACM range from small panels of specific diseases to exome and genome sequencing. The more extensive the search, the greater the risk of identifying variants of uncertain significance that make it more difficult to interpret the results. However, advances in understanding of the genetic underpinnings of inherited cardiomyopathies have brought new possibilities for interventions. This is driving a new imperative to elucidate the nuanced ways in which individual combinations of genetic variation, comorbidities, and lifestyle may influence cardiomyopathy phenotypes [36, 38].

The observation that a disproportionate number of ACM patients are athletes led to the hypothesis that physical exercise may be a risk factor for disease development and progression with an incremental effect [39]. In a group of 47 athletes with a definite or probable diagnosis of ARVC, 41 of them practiced endurance sports [40]. Furthermore, desmosomal mutations were found in only six patients, of which two had a family history of ARVC. In addition, the higher the exercise load, the lower the rate of desmosomal mutations. So, it was inferred that high-intensity exercise could mimic the ARVC phenotype even in subjects with no known mutations [41, 42, 43, 44]. Ruwald *et al*. [45] evaluated via questionnaire the relationship between sport, age of its onset and arrhythmia risk in 108 probands. They noticed that those who took part in competitive sports had a lower age at diagnosis and higher risk of ventricular arrhythmias/SCD compared to those who were inactive or engaged in recreational activities. Later studies confirmed the results and added further knowledge. The John Hopkins group evaluated the safety of AHA minimum recommended physical activity in healthy carriers of desmosomal mutations [46]. To do so, they interviewed 28 relatives of 10 probands. Healthy carriers who restricted physical exercise to minimum recommended had no arrhythmic events. On the other hand, probands were found to have undergone a more intensive exercise load than their relatives; a later observation by the same group showed that the arrhythmic risk is dramatically reduced by exercise restriction once the diagnosis is made.

Male ACM patients have a higher arrhythmic risk compared to females. This may be due to the propensity of males to engage heavier in sports. Hormonal influence may play a role too: androgenic hormones trigger adipogenesis, which can enhance disease progression and may also explain why ACM tends to manifest between the twenties and the forties, being exceedingly rare before puberty [47].

**Fig. 4.**
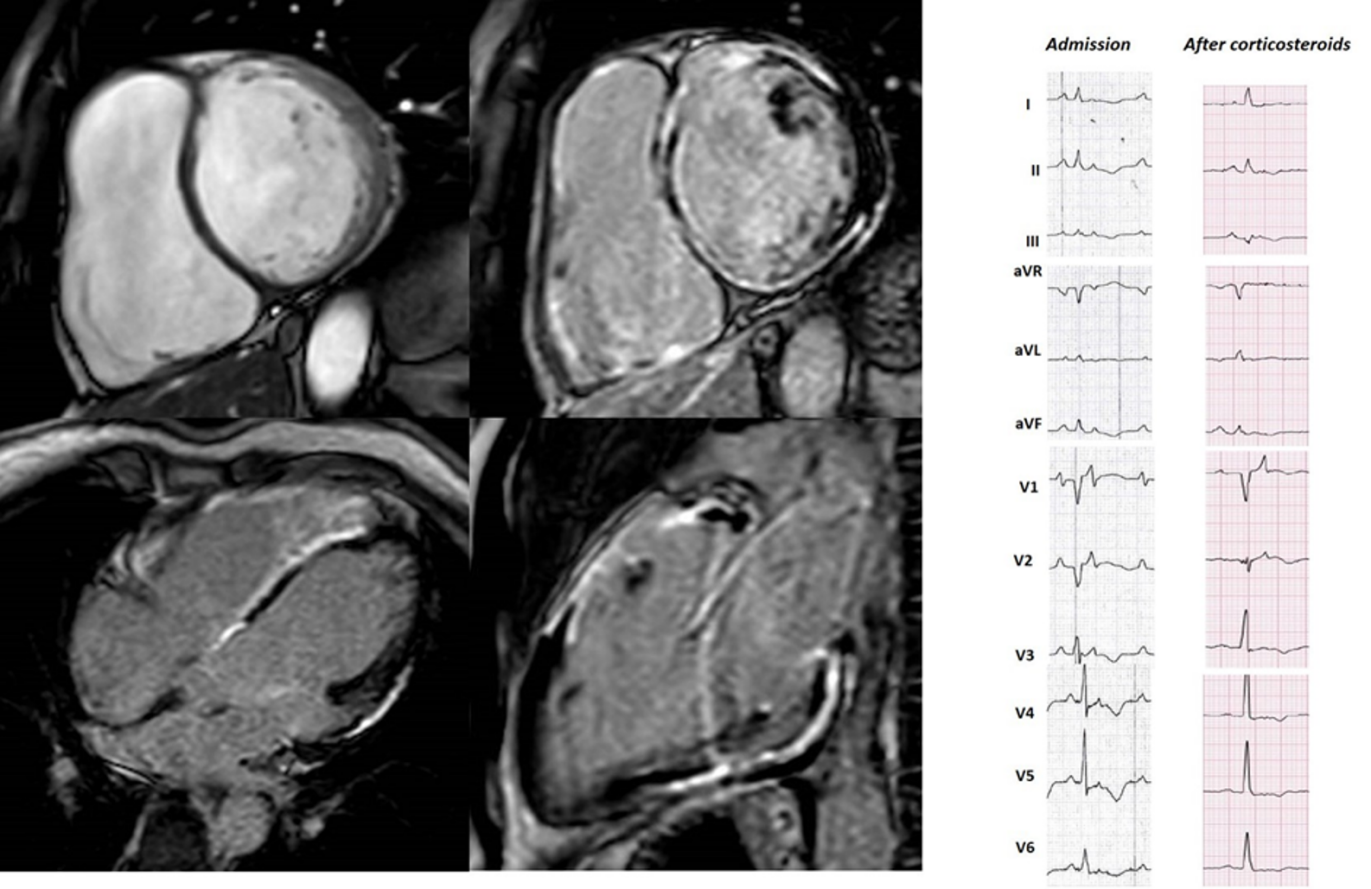
**48 years old male, endurance sportsman, with active sarcoidosis admitted in class 4 heart failure with recurrent slow monomorphic VTs**. The ECG at admission mimics ARVC with a caricatural delayed high-amplitude epsilon wave and precordial T-wave inversion resulting in QT prolongation. Clinical improvement and partial ECG modification occurred after 30 days of steroid treatment. CMR showed biventricular enlargement and systolic dysfunction, with biventricular epicardial and midventricular LGE distribution. This case highlights the diagnostic challenges in ACM.

The observation of inflammatory infiltrates in up to two-thirds of autoptic ARVC diagnosis implies that inflammation may have a role in ACM pathogenesis [48]. Protonotarios *et al*. [49] retrospectively analysed 16 ARVC patients referred for 18F-fluorodeoxyglucose positron emission tomography (FDG-PET)—a validated technique for detecting myocardial inflammation in suspected myocarditis. Despite a few study limitations, the group proved 36% of their ARVC patients on FDG-PET had active myocardial inflammation. Inflammatory cytokines have been found at higher levels in the serum of ARVC patients. In addition, there is gathering evidence of autoimmunity [50] being involved, as inferred by the identification of anti-desmosome antibodies that could be produced by the unmasking of epitopes caused by the disease. Indeed, a myocarditis-like clinical presentation is increasingly reported in ACM patients, either being the initial trigger of the disease or a transient “hot phase” along its clinical continuum [51]. To further complicate things, there is clinical and pathological overlap between ACM and sarcoidosis, another inflammatory myocardial disease (Fig. 4) [52, 53]. Thus, ACM diagnosis remains clinical challenging both in the early stages when myopericarditis, sarcoidosis, or other inflammatory disease need to be ruled out, and at later stages when ischemic/nonischemic dilated cardiomyopathies are concerned.

## 3. Diagnosis

### 3.1 The Complexity of Diagnosis

The first detailed pathological description of arrhythmogenic right ventricular cardiomyopathy (ARVC) dates back to the XIX century treatise “*De l’auscultation médiate ou traité du diagnostic des maladies des poumons et du Coeur”*, describing the association of myocardial fat accumulation and right ventricular wall thinning with sudden death. Although this condition has been known for a long time, the diagnosis of ARVC still constitutes a challenge owing to the phenotypic overlap with other causes of right ventricular enlargement or dysfunction such as pre-mitral left-to-right shunts and cardiac sarcoidosis [53]. ARVC diagnosis is mainly suspected based on its phenotypic expression rather than on its genotype, so a subset of clinical features were identified as disease markers. In 1994, a first ARVC diagnostic score was developed based on qualitative parameters including family history, ECG abnormalities, arrhythmic events, and cardiac functional and structural abnormalities. The imaging diagnostic criteria gained by echocardiography, cardiac magnetic resonance (CMR), invasive cardiac angiography (ICA) or nuclear imaging focused on the evidence of RV dilation, reduced RV systolic function (in the absence of or with only mild LV impairment), regional RV motion abnormalities (i.e., small aneurysms, akinetic or dyskinetic areas with diastolic bulging). When addressing the ARVC diagnostic pathway, myocardial fibro-fatty replacement at biopsy (major criterion) was not considered sufficient to diagnose ARVC as this finding can be observed in several other conditions. Furthermore, given the typical segmental pattern of fibro-fatty replacement in ARVC, the histological diagnosis is burdened by a low sensitivity. However, it retains a high clinical value when alternative diagnostic hypotheses like sarcoidosis or myocarditis are considered.

Electrocardiographic criteria for right ventricular involvement included relatively specific depolarization abnormalities such as: (1) epsilon waves (small depolarization signal between the end of the QRS complex and the beginning of the T wave) and QRS prolongation in right precordial leads (Major criteria); (2) late potentials on signal-averaged ECG; (3) repolarization abnormalities like isolated T wave inversions in right precordial leads; (4) left bundle branch block (LBBB) type ventricular tachycardia, and frequent ventricular extrasystoles (>10,000/24 h). Lastly, histological confirmation of the disease in a close relative was considered a major criterion [54].

Given the high specificity but low sensitivity of these criteria, they were revisited in 2010 with the addition of genetic and quantitative structural and functional parameters. In particular, the evidence of pathogenic variants in ARVC-related genes was introduced as a major diagnostic criterion [55]. Despite these additions, a 2020 review paper by a group of international experts outlined a number of shortcomings of the current diagnostic system. Firstly, the 2010 criteria had been developed for those cases with exclusive RV involvement and therefore cannot aid the diagnosis of left dominant and biventricular forms. Secondly, the role of genetic analysis, introduced in the 2010 diagnostic score as a major criterion, was questioned given the high uncertainty surrounding several genetic variants linked to ACM as these are often non-specific and can also be found in healthy subjects. Eventually, despite the introduction of morphological and functional CMR features in the 2010 criteria, tissue characterization was not included [56].

The 2020 development of the “Padua Criteria” aimed at overcoming precisely these diagnostic weaknesses by including left ventricular diagnostic parameters. These criteria keep the previous score system’s outline based on major and minor diagnostic criteria divided into six groups (morphological and functional ventricular abnormalities, structural myocardial abnormalities, repolarization abnormalities, depolarization abnormalities, ventricular arrhythmias, family history and genetics) [57]. According to this update, ARVC diagnosis should be regarded as certain in the presence of two major criteria, or one major and two minor criteria, probable in the presence of one major and one minor criterion or three minor criteria from different categories, and possible in the presence of one major or two minor criteria from different categories.

ALVC is on the contrary diagnosed in the presence of* one structural criterion* for left-ventricle (LV) involvement (with or without the association of morphological and functional abnormalities)* and* the demonstration of *an associated genetic mutation*, in the absence of RV involvement. Based on the histologic/CMR correlations observed in ARVC, the clinical entity of LV fibro-fatty involvement at the epicardial/midventricular layers coupled with ventricular arrhythmias and with varying degrees of LV dysfunction is currently investigated as a suspected ALVC in a view to determine its possible genetic basis.

ECG abnormalities for ALVC are nonspecific and include inverted T waves in left precordial leads (V4–V6) in the absence of complete LBBB, low voltage QRS in limb leads and ventricular arrhythmias with right bundle branch block (RBBB) morphology (Table 1).

**Table 1.** ** Main ECG abnormalities in ACM**.

	RV involvement	LV involvement
Depolarization abnormalities	Right precodial leads:	Low voltage QRS in limb leads (<0.5 mV from peak to peak).
	- Εpsilon wave and late potentials on signal-averaged ECG;	
	- QRS prolongation with terminal S wave delay (>55 ms).	
Repolarization abnormalities	T wave inversion in right precordial leads (V1–V3).	T wave inversion in left precordial leads (V4–V6).
Ventricular arrhythmias	VPCs, NSVTs, SVTs with LBBB morphology.	VPCs, NSVTs, SVTs with RBBB morphology.

RV, right ventricle; LV, left ventricle; VPC, ventricular premature complex; NSVT, non-sustained ventricular tachycardia; SVT, sustained ventricular tachycardia; LBBB, left bundle branch block; RBBB right bundle branch block.

Finally, to diagnose biventricular forms of ACM at least one morphological/functional or structural criterion for both the right and the left ventricle are needed [57]. As can be noted, ARVC diagnosis is mainly based on phenotype and clinical manifestations, whereas *genotype assessment* plays a predominant role in ALVC diagnosis, as many etiologies/genetic mutations can subtend a similar phenotype. So, ARVC and ALVC can hardly be considered as the same disease with different clinical presentations, given the broad spectrum of manifestations and the different genetic alterations supposed to be disease-causative.

A further source of complexity emerges from the 2019 HRS consensus definition of ACM, extending the definition of ACM to all types of arrhythmogenic heart muscle disorders that are not explained by ischemic, hypertensive, or valvular aetiologies [58].

The pitfalls of previous diagnostic scores and the limited adoption of Padua criteria to date, as well as the nosological ambiguity surrounding ACM, strongly demand a shared diagnostic approach to this uncharted field of cardiology.

### 3.2 Imaging

Imaging techniques play a fundamental role in the diagnosis and follow-up of ACM patients. Indeed, these are not limited to the assessment of morphological and functional abnormalities of the heart, but extend also to tissue characterisation and in particular to myocardial fibro-fatty replacement. Nowadays, echocardiography and Cardiac MRI (CMR) are the most important techniques, while right ventriculography is outdated.

#### 3.2.1 Echocardiography

Despite the advances in the field of echocardiography and CMR, these techniques are still fraught with significant inter-observer variability, especially regarding the functional evaluation of the right ventricle. Echocardiography is usually the first employed in suspect cases, allowing quantitative measurements of ventricular dilation, global systolic function, and regional wall motion abnormalities of both the right and the left ventricle. The 2010 Task force criteria (TFC) included RV akinesia, dyskinesia, or aneurysm together with RVOT diameter (in PLAX or PSAX) and fractional area change, as echocardiographic parameters to screen ARVC. These were shown to be highly specific for ARVC, but to lack sufficient sensitivity, especially during the early disease stages. Other structural findings, such as right ventricular trabeculae and moderator band thickening, are less specific as these can also be found in healthy athletes. Furthermore, in 2017 a consensus by the European Association of Cardiovascular Imaging (EACVI) recommended the assessment of tricuspid annular plane systolic excursion (TAPSE), RV basal diameter and TDI tricuspid lateral annulus peak systolic velocity (s’). The diagnostic performance of echocardiography in ARVC may be increased by speckle tracking imaging (STE) and 3D imaging, as strain analysis of myocardial regional deformation may provide further increased diagnostic accuracy as well as evidence of early myocardial involvement. Several studies have shown reduced global and longitudinal RV strain in patients with ARCV [59, 60]. Moreover, Mast *et al*. [61] observed that in relatives of ARVC patients normal sub-tricuspid regional strain is associated with no disease progression in the next 4 years follow-up, strain abnormalities possibly anticipating the overt signs of the disease. To date there are only a few reports describing LV strain abnormalities in the setting of ACM [62]. 3D echocardiography enables a more accurate measurement of RV volumes and ejection fraction, thereby overcoming the geometrical limits of 2D RV imaging, that underestimates RV volumes. 3D echocardiography measurements correlate well with CMR corresponding values [63]; RV function should be considered abnormal when the EF is lower than 40–45% [64, 65]. However, the main limitations of these imaging modalities are the need of highly skilled technical expertise, the potential acquisition challenges in the presence of ventricular ectopic beats and of severely enlarged RV during late stages of disease, and the absence of reference values for ACM patients. It should be also noted that while RV volumes are increased in the late stages of ACM, they are usually in the normal range in the early stages [66].

#### 3.2.2 Cardiac Magnetic Resonance

*Cardiac Magnetic Resonance* (CMR) should be considered as a third level imaging technique. Owing to its high spatial and temporal resolution, it is the gold standard for the evaluation of biventricular morphology, volumes, wall thickness, mass, and global and regional systolic function. Its added value is represented by the potential for tissue characterization and in particular for fibro-fatty infiltration. Indeed, CMR tissue assessment constitutes one of the most important additions in the recently developed Padua Criteria [57]. It should also be noted that CMR abnormalities are not sufficient for a definite diagnosis of ACM and that the diagnosis is rather the result of the combination of various features including family history, ECG features, arrhythmic events, imaging, and histological data. In keeping with this notion, patients with ACM rarely present CMR abnormalities without either ECG or Holter ECG abnormalities [67]. Importantly, CMR plays a pivotal role in the differential diagnosis of ACM phenocopies as fibro-fatty replacement can be found in the setting of other diseases as well (e.g., ischemic cardiomyopathy, myocarditis, sarcoidosis), whose location and extent of fibro-fatty replacement is different from ACM. Furthermore, the identification of adipose tissue (typically located in the subepicardic/intramyocardial regions of the ventricles) carries prognostic value even in the setting of normal ventricular volume and function. Indeed, a recent study by Aquaro *et al*. [68] demonstrated that in 175 ARVC patients (52 definite diagnosis, 50 borderline diagnosis, 73 possible diagnosis) the presence of fat tissue infiltration represented an independent predictor for adverse events in the global population (HR 3.69, 95% CI 1.57–8.65, *p* = 0.0002) as well as in the group of patients with a definite ARVC diagnosis (HR 3.03, 95% CI 1.15–8.02, *p* = 0.02).

## 4. Treatment

Once a definite diagnosis is established or is probable, patients should undergo a thorough evaluation and management that includes arrhythmic risk stratification and prevention of SCD, management of recurrent ventricular arrhythmias, treatment of progressive myocardial dysfunction and familial screening.

ICD implantation is the only proven therapy that has a significant impact on mortality. However, it does not come without possible early and/or late complications, considering that most ACM are young active adults. While there is shared agreement on ICD implantation for secondary prevention after cardiac arrest due to VF/VT and sustained ventricular tachycardia with or without hemodynamic compromise, there is no definite consensus for primary prevention of SCD. ACM patients have an increased risk of SCD compared to the general population but quantifying this risk at the individual level is difficult. Currently available risk scoring systems are nearly totally based on data taken from retrospective observational studies or on prediction of proper ICD therapies as a surrogate of SCD, though not every ICD-interrupted arrhythmia would have resulted in cardiac arrest [69]. Furthermore, the type and percentage of mutations known to be associated with higher SCD risk vary among these studies.

The International Task Force Criteria [70], proposed in 2015, stratified patients in:

⋅ High risk: aborted SCD, sustained VT, severe RV or LV dysfunction (COR I).

⋅ Intermediate risk: non sustained VT, unexplained syncope, moderate RV or LV dysfunction (COR IIa); male sex, proband status, T wave inversions on ≥3 precordial leads, arrhythmia inducibility at EPS (COR IIb).

⋅ Low risk: healthy carriers with no risk factors (COR III).

In 2019 new recommendations for ICD implantation from the Heart Rhythm Society [58] were proposed. These guidelines are based on the HRS ACM definition, which includes every arrhythmogenic heart disease not due to ischemic, valvular or hypertensive aetiology. This means that even non-inherited diseases (i.e., Chagas, sarcoidosis) are included. With respect to ITFC, these recommendations provide indications for ICD implantation in carriers of some, but not all, genetic mutations (lamin A/C, filamin C and phospholamban).

Both ITFC and HRS divide patients into risk categories, but do not provide a quantitative estimate of their arrhythmic risk. In 2019 a new risk scoring system was proposed by a joint committee based on retrospective data from five centres across North America and Europe [71]. The registries made up a population of 528 patients with a definite ARVC by 2010 Task Force Criteria and no history of VA/cardiac arrest prior to diagnosis. The model was built on pre-specified predictors (sex, age, recent syncope, NSVT, 24 h PVC count, number of anterior and inferior leads with T wave inversion, RV ejection fraction) which yield a quantitative 5-year risk of VA. This risk scoring system accurately predicted 5-year event free survival and outperformed ITFC recommendations on ICD indication: compared to ITFC’s the new system would have resulted in 20.6% less ICD placements, while protecting the same number of patients from VA. Though probably better, this scoring system needs validation and cannot be applied to every population. In 2021 the same group created a new model to estimate individual life-threatening arrhythmia risk [72]. Notably, prior sustained VA did not predict potentially lethal events.

The electrophysiological study (EPS) is a potential tool for risk-stratification as suggested by the 2019 HRS expert consensus statement, though sometimes underused in clinical practice owing to its variable sensitivity and reproducibility [58]. Moreover, electroanatomical mapping may also be a potential marker of an arrhythmic substrate, unveiling fragmented electrograms and endocardial low-voltage area were associated with scar burden and arrhythmic events [73, 74]. Multiple studies have reported that sustained ventricular arrhythmia during programmed ventricular stimulation are prognostic markers of future events [75, 76, 77, 78]. However, an electrophysiological study-based approach in all ACM patients would suffer the risk of either overtreatment in patients with a silent substrate, or undertreatment of non-inducible patients. To this end, it seems useful to integrate EPS in a two-step multifactorial approach with noninvasive findings leading to programmed ventricular stimulation. A similar approach has already been adopted for high risk post-myocardial infarction (MI) patients with an LVEF >40% [79] and is now under evaluation among patients with dilated cardiomyopathy with either relatively preserved (35% < EF > 50%) or reduced (LVEF <35%) systolic function [80].

Recent studies have focused on a totally different approach, that is SD risk assessment of genotyped subjects based on LV dysfunction. A study conducted in lamin A/C mutation carriers, who either had a left-dominant ACM or DCM phenotype, identified a left ventricle ejection fraction below 45% as a factor for increased arrhythmic risk [81]. Similar results were obtained in a population of filamin C carriers.37 Similarly, in phospholamban p.Arg14del carriers, left ventricle ejection fraction below 45% and a personal story of sustained and non-sustained ventricular tachycardias were both associated with an increased risk of ventricular arrhythmias [82]. A recent study on phospholamban mutation carriers issued in 2021 implemented the previous model of risk stratification by introducing specific characteristics of left ventricular involvement as low-voltage QRS and T wave inversion [83]. The ignorance of genotype abnormalities in the general population limits their assessment as reliable independent prognostic markers for a SCD prevention strategy.

To complicate things, there is no shared consensus on the type of ICD to implant (transvenous vs subcutaneous). Each has specific advantages (longer battery life, possibility of ATP and anti-bradycardia pacing for transvenous; lower risk of lead malfunction and endocarditis for subcutaneous) and drawbacks (transvenous: lead malfunction/fracture, risk of endocarditis, pneumothorax; subcutaneous: higher rate of inappropriate shocks, lower battery life, no possibility of ATP/bradycardia pacing) [84]. Choice often depends on implanter preferences beyond patients’ profile (i.e., age, Lamin A/C mutation and risk of bradyarrhythmias).

Besides ICD placement, disease progression should be prevented and defibrillator therapies reduced. Physical exercise restriction has a major impact on both aspects, as aforementioned. β-blockers are often prescribed as arrhythmia in ACM are often triggered by increased adrenergic drive, though this is not an evidence-based approach. Antiarrhythmic drugs, mainly amiodarone and sotalol, are used as a second line. Drug-refractory arrhythmias can be treated by transcatheter ablation, which has achieved a good success rate thanks to combined endocardial-epicardial approach [85]. However, ablation has no demonstrated impact on survival and carries risks [86, 87]. Patients that progress to ventricular dysfunction are treated according to heart failure guidelines, though evidence-based efficacy of drugs for HFrEF is lacking in this setting.

## 5. Single Centre Experience

### 5.1 Materials and Methods

This study is a retrospective analysis of the data of consecutive patients with confirmed diagnosis of ACM based on the proposed Padua Criteria, who were referred to our centre from January 1992 to October 2021. The aim of this study was to identify characteristics of ventricular arrhythmias and treatment in patients with ACM.

Clinical information regarding demographics, symptoms, 12-lead ECG, echocardiogram, CMR, and genetics were collected. In addition, data regarding ICD therapies and arrhythmia occurrence were obtained for each patient. Decisions regarding ICD programming were made by the managing cardiologist and/or electrophysiologist, namely VF+ single VT zone (conditional shocking zone for S-ICD), or VF + 2 VT zones based on available clinical data. Ventricular tachycardia (VT) detection was programmed at a cutoff rate as 171 bpm for a duration of 20–25 seconds, while VF detection was set at 231 bpm for a duration of at least 9 seconds, arrhythmia discriminators turned ON at their best possible performance [88, 89, 90, 91, 92]. When a slow VT zone was programmed in the range 120–170 bpm, detection was at least 35 seconds; shock therapy in this zone was programmed only after the arrhythmia had proved to cause severe hypotension or cardiogenic shock.

Anti-tachycardia pacing (ATP), either as Burst (minimum 2 attempts) and Ramp (minimum 1 attempt) pacing, was programmed as first delivered therapy in the VT zone, whereas it was delivered either before or during shock charging in the VF zone (according to each manufacturer specificity), while shock therapy was available in both VT and VF zone. Arrhythmia history and delivered therapy were analyzed either at in-clinic and at remote patients’ follow-ups. Clinical assessment, drugs and antiarrhythmic drug prescription were evaluated at twice yearly follow-up unless more frequent examinations were deemed necessary. Arrhythmia analysis was carried out by 5 experienced electrophysiologists based on stored intra-cardiac electrograms (EGMs).

Ventricular arrhythmia (VA) was defined as a regular or irregular ventricular tachycardia at cycle length <430 ms; life-threatening ventricular arrhythmia (LT-VA) was defined as an irregular or regular tachycardia with a mean cycle length (CL) of ≤300 ms. Appropriate ICD intervention was defined as an ICD therapy for VA/LT-VA. An inappropriate intervention was defined as therapy delivery because of supraventricular tachycardia or oversensing due to either cardiac or non-cardiac signals.

### 5.2 Statistical Analysis

Continuous variables are summarized as either mean ± SD or median (interquartile range) and compared across groups using a Mann–Whitney or Kruskal-Wallis test. Categorical variables are reported as frequency (percentage) and compared between groups by a χ^2^ or Fisher exact test. The cumulative probability of survival free from first appropriate ICD intervention (VA/LT-VA) and from intervention for VA/LT-VA was determined by the Kaplan–Meier method, and differences in survival between groups were evaluated with the log-rank test. In patients without an ICD intervention, follow-up was to the most recent evaluation, transplantation, or date of death, whichever came first. All analyses were performed using SPSS 23.0 (SPSS Statistics/IBM Corp, Chicago IL, USA). A *p *≤ 0.05 was considered significant.

## 6. Results

### 6.1 Patient Population

The patient population consisted of 72 patients with diagnosis of ACM, confirmed using retrospectively the proposed Padua Criteria. Of these, 29 patients (40.3%) were initially diagnosed using 1994 ITF Diagnostic Criteria for ARVC and 18 (25%) using the 2010 Proposed Modification of the Task Force Criteria. Twenty-two (30.6%) patients with exclusive left ventricle involvement were diagnosed as ALVC according to the characteristic ring-like LGE LV pattern at CMR associated genetic mutation and/or familial history of AC and/or red flags for ALVC (i.e., negative T waves in V4-6/aVL, low voltages in limb leads, right bundle branch block-like ventricular tachycardia) or defined on microscopic analysis in explanted heart examinations. Three (4.1%) patients were diagnosed after the introduction of Padua Criteria.

At the time of ICD implant, 29 patients (40.3%) had right ventricular involvement, 24 (33.3%) had a dominant LV involvement, and 19 (26.4%) had biventricular involvement. During follow-up, 6 ARVC patients and 2 ALVC patients evolved to a biventricular pattern.

The mean age at implant was 46 ± 16 years; 48 patients (66%) were males, 68/72 implanted at our centre. The patients were followed for a median follow-up of 6.1 years [IQR: 2.5–9.9], genetic testing was performed in 45 patients (62.5%) and a pathogenic mutation was observed in 35 (80%) of these patients. The genes most frequently involved were desmoplakin (41.7%) and plakophillin2 (22.2%); 7 patients (20%) had more than one mutation.

Following the diagnostic criteria themselves, and the more recent enrolment, patients with ALVC had a better genetic characterization, compared to ARVC and biventricular ACM.

Population characteristics at implant are described in Table 2.

**Table 2.** **Patients characteristics at implant**.

		ACM (n° 72)	ARVC (n° 29)	ALVC (n° 24)	Biventricular ACM (n° 19)	*p*-value
Clinical characteristics at implant						
	Mean follow-up ± SD (years)	7.0 ± 6.1	8.5 ± 6.6	5.1 ± 4.5	7.3 ± 6.3	0.2
	Median follow-up ± IQR (years)	6.1 [2.5; 9.9]	6.1 [2.7; 13.0]	4.2 [1.6; 7.8]	6.13 [2.9; 10.1]	
	Male	48 (66.6%)	21 (72.4%)	15 (62.5%)	12 (63.2%)	0.7
	Mean age at implant, years	46 ± 16.3	50.0 ± 15.3	42.7 ± 16.4	44.5 ± 17.3	0.27
	Family history of SD	35 (48.6%)	9 (31%)	17 (71%)	9 (47.4%)	**0.02**
	Family history of ACM	23 (31.9%)	8 (27.6%)	11 (45.8%)	4 (21.1%)	0.18
	Syncope	26 (36.1%)	14 (48.2%)	3 (12.5%)	9 (47.4%)	**0.01**
	Cardiac arrest	8 (11.1%)	4 (13.8%)	3 (12.5%)	1 (5.3%)	0.72
	Ventricular arrhythmia	23 (31.9%)	12 (41.4%)	4 (16.7%)	7 (36.8%)	0.19
Genetic analysis		n° 45	n° 10	n° 24	n° 11	
	Mutation carrier	36 (80.0%)	5 (50%)	24 (100%)	7 (63.6%)	< **0.01**
	DSP mutation	15/36 (41.6%)	1/5 (20%)	11/24 (45.8%)	3/11 (27.3%)	< **0.01**
	PKP2 mutation	8/36 (22.2%)	4/5 (80%)	0	4/11 (36.4%)	< **0.01**
Antiarrhythmic drugs		53 (73.6%)	20 (68.9%)	15 (62.5%)	19 (100%)	
	Amiodarone	6 (8.6%)	1 (3.4%)	3 (12.5%)	2 (10.5%)	0.49
	Sotalol	11 (15.3%)	8 (27.6%)	1 (4.2%)	2 (10.5%)	0.05
	Flecainide	0	0	0	0	
	Propafenone	0	0	0	0	
	Beta-blockers	39 (54.2%)	11 (37.9%)	13 (54.2%)	15 (78.9%)	0.013
Ecocardiography		n° 69	n° 27	n° 24	n° 18	
	Area RV td, ${\mathrm{cm}}^{2}$	27.6 ± 8.4	28.1 ± 9.2	22.7 ± 5.5	29.38 ± 8.0	0.3
	FAC, %	32 ± 16	30 ± 10	46 ± 14	28 ± 7	<0.01
	Vol Vsn td, mL/$m^{2}$	62.8 ± 9.0	55.2 ± 17.6	64.3 ± 15.5	72.3 ± 21.6	**0.02**
	FE Vsn, %	51 ± 13	57 ± 10	51 ± 10	43 ± 14	< **0.01**
	CARDIAC MR	n° 54	n° 18	n° 23	n° 13	
	LGE	36 (66.6%)	6 (33.3%)	23 (100%)	9 (69.2%)	< **0.01**
	Vol TD Vdx, mL/$m^{2}$	106 ± 48	137 ± 57	78.3 ± 14.8	124 ± 54	< **0.01**
	FE Vdx, %	47 ± 13	40 ± 15	56 ± 6	37 ± 11	< **0.01**
	FE Vsn, %	50 ± 10	53 ± 11	52 ± 9	43 ± 11	0.051
	HOLTER ECG 24 h	n° 56	n° 19	n° 23	n° 14	
	Non sustained VT	25 (44.6%)	10 (52.6%)	9 (39.1%)	8 (57.1%)	0.7
	ECG	n° 67	n° 25	n° 24	n° 18	
	ε wave	9 (17%)	3 (12%)	2(8.3%)	4 (22%)	0.45
	Inverted T waves in ≥3 precordial leads	19 (28%)	10 (40%)	1 (4.2%)	8 (44%)	< **0.01**
ICD characteristics						
	Primary prevention	46 (63.9%)	16 (54.2%)	19 (79.2%)	11 (57.9%)	0.21
	Secondary prevention	26 (36.1%)	13 (44.8%)	5 (20.8%)	8 (42.1%)	
	Single chamber	39 (54.2%)	15 (51.7%)	10 (41.7%)	14 (73.7%)	<0.01
	Double chamber	9 (12,5%)	7 (24.1%)	1 (4.2%)	1 (5.3%)	
	CRT-D	3 (4.2%)	1 (3.4%)	0 (0%)	2 10.5%)	
	S-ICD	18 (25.0%)	5 (17.2%)	11 (45.8%)	2 (10.5%)	
	Extravascular	2 (2.8%)	0 (0%)	2 (8.3%)	0 (0%)	
	Epicardic	1 (1.4%)	1 (3.4%)	0 (0%)	0 (0%)	

### 6.2 ICD Implantation

Primary prevention devices were implanted in 46 patients (63.9%), whereas 26 (36.1%) received a device for secondary prevention of SCD. A transvenous ICD was implanted in 51 patients (70.8%); single chamber ICD was the most frequent (39; 54.2%). Only 3 patients (4.2%) received a CRT-D, but during follow-up there were 3 up-grades from single-chamber to CRT-D. Eighteen patients (25%) underwent implantation of a subcutaneous ICD, 1 patient had an epicardial ICD (1.4%), and 2 patients (2.8%) received an extravascular ICD.

### 6.3 Appropriate ICD Therapy

**Fig. 5.**
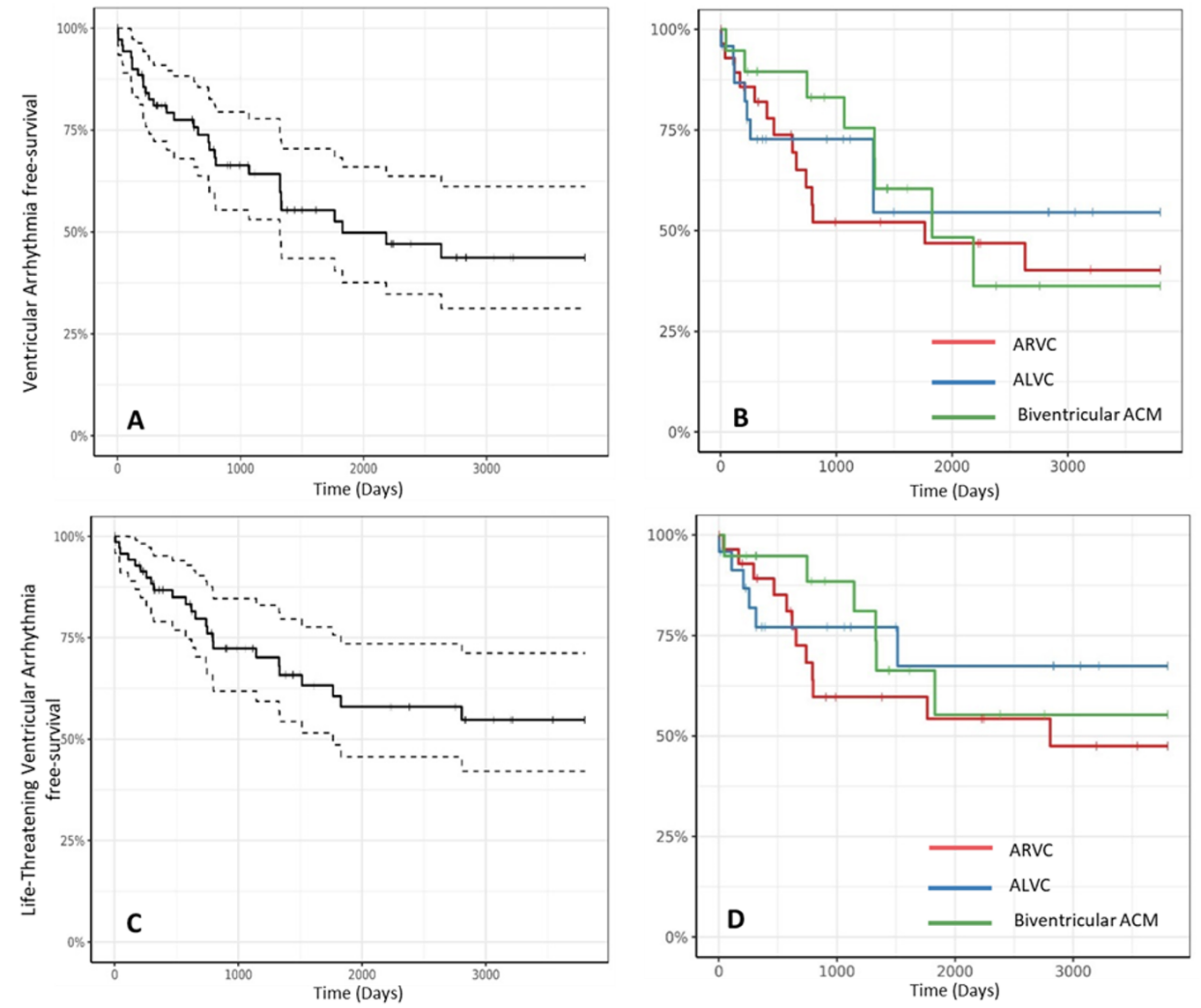
**Freedom from ventricular arrhythmias requiring ICD therapy delivery (A,B) and from life-threatening ventricular arrhythmias requiring ICD therapy delivery (C,D)**.

**Fig. 6.**
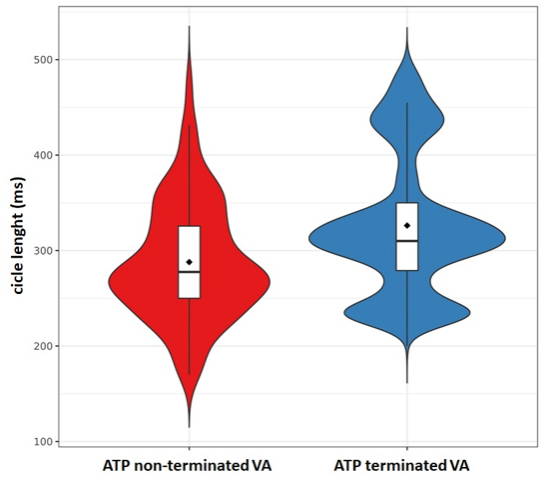
**Cycle length and distribution of ATP-terminated vs non-terminated Ventricular Arrhythmias (VA)**.

During follow-up, 34 patients (47.2%) had ventricular arrhythmias treated by the ICD. Fig. 5 shows the Kaplan–Meier analysis of cumulative survival from first appropriate ICD therapy on VA and LT-VA. Overall, the cumulative survival free from appropriate ICD interventions was 81%, 64% and 53% at 1, 2 and 5 years, respectively. Considering only life-threatening events (cycle length ≤300 ms), 27 patients (37.5%) received appropriate therapy. Overall, the cumulative survival free from appropriate ICD interventions on LT-VA was 87%, 72% and 61% at 1, 2 and 5 years, respectively (Fig. 5). Within the three phenotypic variants (ARVC, ALVC and biventricular) there were no significant differences in the incidence of appropriate ICD intervention on VA or LT-VA (Fig. 5). ICD intervention characteristics are described in Table 3. The 34 patients who received an appropriate ICD activation had 919 therapies delivered because of VA, in total. Of these 34 patients, 27 had 314 episodes of LT-VA. The mean cycle length of LT-VA was 248 ± 25 ms. LT-VA was the first arrhythmic episode treated by ICD in 18 patients (52.6%). Of the 919 VA, 914/919 (99.4%) occurred in patients with an ICD capable of delivering ATP, while 5 (0.6%) occurred in s-ICD recipients. Considering only the 32 patients with an ICD capable of delivering ATP, 735 VA (80.4%) were successfully terminated with ATP; 179 VA (18.6%) did not respond to ATP and were treated with a shock. Of the 309 episodes of LT-VA, 201 (65%) of the 309 episodes of LT-VA (65%) were terminated by ATP and 108 (35%) by a shock, respectively. In 29/32 patients (91%) ATP terminated at least one episode of VA and in 14/25 (56%) at least one episode of LT-VA. The median cycle length of ATP-terminated vs non-terminated VA was respectively 310 [279–350] vs 278 [250–326] ms (*p *< 0.001); the distribution is reported in Fig. 6. Two patients in the end-stage of biventricular ACM had a slow VT zone programmed to treat monomorphic VAs in the range 120–170 bpm recurrent despite ablation, which were managed by drug therapy and ATP. Shock was disabled in this VT zone. VT ablation was carried out in four patients with ARVC (3 endocardial, 1 epi/endocardial), 3 of them having recurrences at a different cycle length and morphology at follow up: 2 died after 6 and 13 months after ablation. A single ALVC patient with an S-ICD underwent endo-epicardial VT ablation proving non-inducibility, but had 3 VA recurrences in the 190–220 bpm range in the first year, requiring electrical cardioversion; no VAs have been recorded 2 years after the procedure.

**Table 3.** **Appropriate ICD interventions on Ventricular Arrhythmia (VA) and Life-threatening Ventricular arrhythmias classified by ACM phenotype (ARVC, ALVC, Biventricular) and ICD type (ATP capable devices and S-ICD)**.

	ACM (n° 72)			ARVC (n° 29)			ALVC (n° 24)			Biventricular ACM (n° 19)			
Patients with appropriate ICD intervention													
- VA	34 (47.2%)			16 (41.0%)			9 (37.5%)			9 (47.4%)			n.s.
- LT-VA	27 (37.5%)			13 (33.3%)			7 (29.2%)			7 (36.8%)			
First ICD intervention on LT-VA	18			9			5			4			n.s.
ATP capable devices	n° 54			n° 24			n° 13			n° 17			
Appropriate intervention on:	**N°**	**ATP**	**Shock**	**N°**	**ATP**	**Shock**	**N°**	**ATP**	**Shock**	**N°**	**ATP**	**Shock**	
- VA	**914**	735	179	**300**	206	94	**427**	401	26	**187**	128	59	n.s.
- LT-VA	**309**	201	108	**78**	26	52	**182**	163	19	**49**	12	37	n.s.
S-ICD	n° 18			n° 5			n° 11			n° 2			
Appropriate intervention on LT-VA	5			2			3			0			n.s.

In S-ICD recipients (18 patients) there were 5 shocks on LT-VA in 2 patients. One had a VA below the detection rate which was treated by electrical cardioversion (CVE) for clinical instability and was treated by ablation as aforementioned.

### 6.4 Inappropriate Therapy Delivery

16 patients (22.2%) had inappropriate ICD therapy, including 10 because of supraventricular arrhythmias (7 atrial fibrillation, 2 sinus tachycardia, 1 atrial tachycardia), 4 because of oversensing (T wave or muscular activity in 2 S-ICD patients, lead noise in 2 transvenous ICD recipients). Nine patients (12.5%) needed reintervention because of loss of ventricular sensing (3 patients), infection (3 cases), insulation defect in a Riata lead (2), lead dislodgement (1 extravascular ICD patient). No re-interventions occurred in the S-ICD subgroup.

### 6.5 Long-Term Outcome

**Fig. 7.**
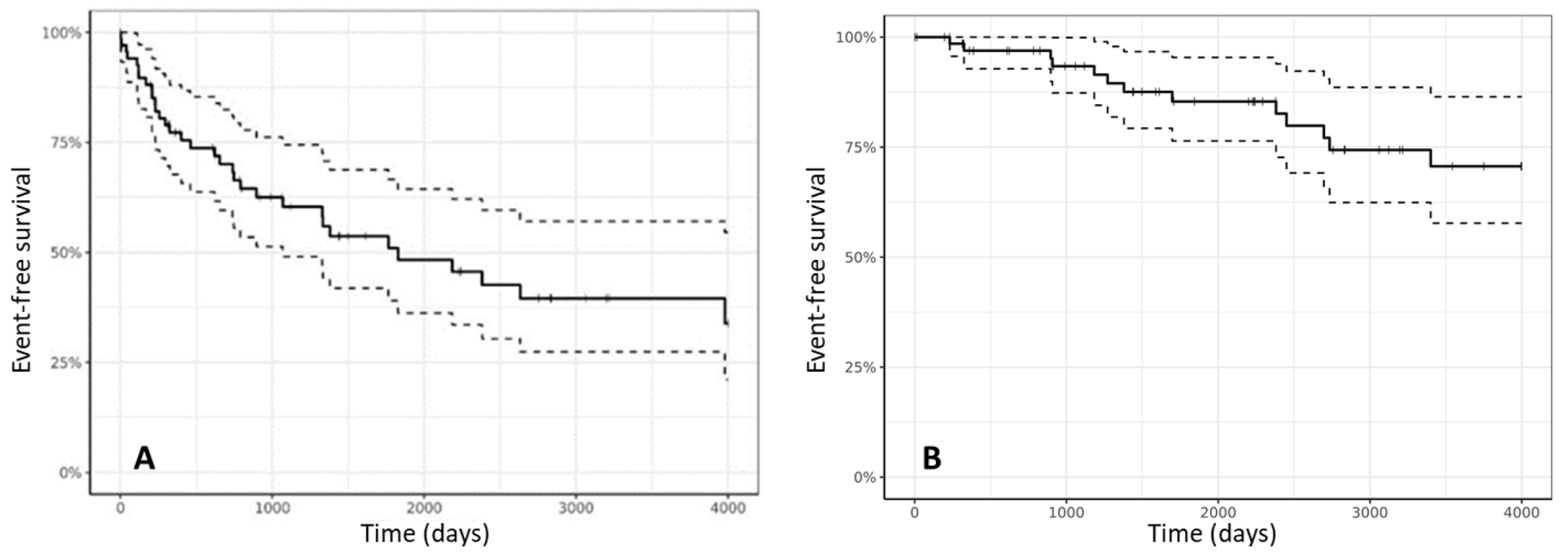
**Freedom from the cumulative end-point of ICD therapy delivery, all-cause death, and heart transplantation (A)**. Freedom from heart failure-related death + heart transplantation (B).

At last follow-up, 61 patients (84.7%) were alive and 11 (15.3%) had died: 7 related to heart failure, 1 for stroke, 3 due to non cardiovascular causes. Eight patients (11.1%) underwent heart transplantation due to refractory heart failure at a relatively young age (Fig. 7A,B). Mean patients age at cardiac transplantation was 38.6 ± 10.8 years. None of them required invasive mechanical assistance before transplant. One patient died over the complications of a CMV donor-driven primary infection.

## 7. Discussion

In this study 47.2% of patients had an appropriate ICD activation during a mean follow-up of 6.1 years. These results are consistent with those reported by similar studies on ACM patients treated by an ICD and with a recent meta-analysis, estimating a 10.6% per year VT incidence [75, 93, 94, 95]. Narrowing it down to life threatening events, which were defined as ventricular arrhythmia with a cycle length ≤300 ms, more than a third of patients presented a LT-VA during follow-up (nearly 5% per year). Fast VAs are considered to be a more reliable index of the risk of sudden cardiac death, however, there is no single definition of fast VA. This distinction is reported in some studies, where cycle lengths cut-offs of 240–250 were mostly used: this would blunt the incidence of LT-VA at 3.3–3.6% per year [75, 96, 97, 98]. However, even fast VA may self-terminate before death [69], so the use of appropriate ICD intervention for fast VA as a surrogate for SCD may lead to an overestimation of the survival benefit from ICD. However, we aimed to avoid the overtreatment of self-terminating VA by programming long detection times (20–25 seconds for VT, 9 seconds for VF).

The population enrolled in this study is a cohort of ICD patients, as in most studies conducted on this topic. The generalizability of these data to an unselected cohort of ACM is therefore limited [94]. However, eliminating the selection bias is difficult, as it is impossible to know the real denominator of the equation, i.e., the number of subjects with ACM. Not knowing the real number of affected subjects, as indirectly demonstrated by the variability in the incidence estimates [99], does not allow us to have an accurate estimate of the arrhythmic risk and probably leads to overestimate it.

Clear and extensive data on the incidence and characteristics of arrhythmias in the different subgroups of ACM is still lacking. In this study there were no significant differences in terms of appropriate ICD intervention on VA or LT-VA within the three phenotypic variants (ARVC, ALVC and biventricular). However, patients with ALVC had a better genetic and instrumental characterization (echocardiogram, CMR) than those with ARVC owing to more widespread CMR availability in recent years. In addition, they were implanted for primary prevention in almost 80% of cases, a fact that may limit the reliability of subgroups comparison.

The population enrolled in this study present a high proportion of DSP mutations compared to other studies where PKP2 is reported to be the most common gene involved [36]. This may be attributed to two factors: on the one hand, patients with DSP mutations have a more severe disease development and are more often associated with arrhythmic events [100], so patients with DSP mutations are more likely to have come to our attention; on the other hand, in our study there is a relatively high frequency of ALVC, and DSP mutations is frequent within this subgroup based on a recent Italian study [101].

Arrhythmic presentation of ACM seems to be age-dependent: while older patients with advanced disease more often experience reentrant VT related to fibro-fatty myocardial scar, in young patients electrical instability prevails, so it is common to observe the abrupt onset of VF [102, 103]. This concept, coupled with the idea that in patients with ACM most “non-fast arrhythmias” are well tolerated and tend to be self-limiting, downsized the role of ATP [104, 105, 106]. Nevertheless, in this study 80.4% of VA and 65% of LT-VA were successfully terminated with ATP. A high success of ATP in terminating arrhythmia was also reported by Link *et al*. [96] and Al Ghamdi *et al*. [107] who found an ATP success rate of 92% and 61%, respectively. Additionally, in the study by Link *et al*. [96] the effectiveness of ATP was independent of the arrhythmia cycle. Regretfully, the detection time in these two studies is unknown, while in our patients it was programmed as to deliver ATP only after 20–25 seconds for VT and after 9 seconds in the VF zone, thus enabling self-termination on non-sustained VA. It is well known from the MADIT-RIT trial [92] and the PAINFREE-SST [89] trial that short detection times lead to overtreatment of non-sustained VA and to overestimation of ATP efficacy, which makes the comparison with our data unreliable. Moreover, ATP programming (number of bursts or ramps) was not specified, as well as ATP delivery for fast VTs in the VF zone, while we used multiple attempts at ATP with both bursts and ramps: this limits the comparison amongst the 3 studies. In the large meta-analysis of more than 6000 patients, Cheng *et al*. [108] observed that ischemic and nonischemic cardiomyopathy patients have similar rates and proportions of monomorphic VT and polymorphic VT/VF episodes, ATP-associated termination of monomorphic VT being comparable between the two groups. As expected, in our patients ATP-terminated VAs had a significantly slower rate compared to ATP-failed VAs, though there was significant rate overlap between successfully ATP-terminated and failed episodes (Fig. 6).

Transcatheter ablation has been shown to be a valuable tool in reducing sustained VAs and ICD activation [86, 109]. However, complications associated with the procedure are not negligible, with a risk of major events such as death and cardiac tamponade between 5–10% [105]. Ablation was not proven to prevent SCD and therefore it cannot be considered a substitute for ICD implantation. Hence, ablation is currently recommended in patients with incessant VT and frequent appropriate ICD interventions despite optimal medical therapy or who do not tolerate medical therapy. Therefore, ATP success in terminating VAs questions the use of ICD not capable of delivering ATP in this young patient population, being associated with good prognosis and quality of life benefits of such a pain-free intervention [92, 110]. Ablation should not be considered an alternative option, but rather complementary to ATP in reducing VAs and ICD interventions [111, 112].

Inappropriate ICD interventions are a serious problem in some patients with ARVC. In our study, 16 patients (22.2%) suffered from inappropriate ICD therapy This finding is consistent with other studies that have reported inappropriate shock rates of between 16% and 27% in ACM patients, who are relatively young [113, 114]. In our study the most frequent complications were related to right ventricular loss of sensing and to infection. A progressive reduction in R-wave amplitude at the intracavitary EGM is a known phenomenon in ACM patients, particularly in ARVC patients, and it is attributed to progressive myocardial fibro-fatty replacement. Low sensing values in the right ventricle may exist already at the time of implantation and progressive decline of signal amplitude together with increased pacing threshold may occur [115, 116, 117]. In this study we reported complications needing reintervention in 9 patients (12.5%), of whom 3 (4.2%) had loss of adequate sensing, despite a strategy to target an RV septal location. As already suggested by some authors, this area may be less affected by fibro-fatty replacement than the apex and free wall of the right ventricle [114, 117]. The S-ICD is perceived to reduce lead-related complications [104, 106]: in this study there were no complications associated with S-ICD, but 2/18 patients reported inappropriate shocks (T-wave double counting and muscular activity oversensing). Nevertheless, similarly to the intracardiac signal, fibro-adipose replacement may lead to a progressive modification of the surface ECG [118, 119]. Data on the impact of ECG evolution on S-ICD sensing are still lacking, particularly for different ACM phenotypes.

During follow-up, 20% of patients who initially had ARVC and 8% of patients with ALVC at the time of implantation progressed to a biventricular involvement. The progressive deterioration of ventricular function and the development of heart failure are a major cause of death in ACM patients, accounting for up to two thirds of deaths [120]. In this study, 15 ICD recipients (21%) died because of heart failure or underwent transplantation. Ventricular dysfunction and heart failure are often recognized lately in ACM patients, particularly in right-sided phenotypes when the classic left-sided signs are absent and fluid overload + poor exercise tolerance appear only at an advanced disease stage [121]. Indeed, owing to improved diagnostic capability and to reduction of arrhythmic sudden cardiac death, survival of ACM is prolonged to the stage when loss of functional myocytes and progressive ventricular dysfunction lead to refractory heart failure. This implies an expected increase of ACM patients in end-stage HF requiring heart transplantation, as anticipated by the different time-course of unexpected SCD and of heart failure-related mortality [1]. ARVC and biventricular ACM patients, due to the right ventricular predominant pathophysiology, require specific considerations for heart failure and heart transplant management: mechanical circulatory support strategies at short and long term are limited in ACM patients, so extreme care must be taken when managing waitlisted patients [122]. ACM patients have good post-transplant course with higher survival compared with other cardiomyopathy aetiologies, owing to the young age with fewer related comorbidities.

## 8. Conclusions

ACM is increasingly diagnosed owing to heightened suspicion at ECG examination and to improved imaging technology and availability, though the diagnostic workflow is particularly challenging in the earliest disease stages. Risk stratification for primary SCD prevention is challenging, especially because neither familial history nor genotyping are reliable risk markers (the prevalence of pathogenic PLN and FLNC mutations being unknown in the general population). ICD is key in SCD prevention, ATP treatment of VA being very effective in this clinical scenario. Disease progression to an advanced stage thanks to SCD prevention very often ends in severe ventricular dysfunction in the fifth to sixth decade, with refractory heart failure that needs to be managed carefully.
